# Combined checkpoint inhibitor therapy causing diabetic ketoacidosis in metastatic melanoma

**DOI:** 10.1186/s40425-017-0303-9

**Published:** 2017-12-19

**Authors:** Pouyan N. Changizzadeh, Shiva Kumar R. Mukkamalla, Vincent A. Armenio

**Affiliations:** 1 Division of Hematology and Oncology, Roger Williams Medical Center, Providence, RI 02908 USA; 2 Boston University School of Medicine, Boston, MA USA

**Keywords:** Nivolumab, Ipilimumab, Dual checkpoint inhibitor therapy, Insulin-dependent diabetes mellitus and diabetic ketoacidosis

## Abstract

**Background:**

There has been a significant improvement in survival of advanced malignancies with the advent of checkpoint inhibitors. These newer treatment modalities come with a wide spectrum of unique side effects, termed immune related adverse events (irAE), ranging from mild skin rash to severe colitis. Included in that spectrum is the rare side effect of autoimmune diabetes mellitus. Despite a few case reports illustrating the incidence of autoimmune diabetes associated with immunotherapy, there has not been much mentioned about exacerbation or acceleration of hyperglycemia in non-autoimmune settings leading to de novo diagnosis of type 2 diabetes mellitus.

**Case presentation:**

We report the case of a 42 year old man with metastatic melanoma and no prior history of diabetes mellitus, who presented with diabetic ketoacidosis (DKA) after 3 cycles of combination checkpoint inhibitor therapy using nivolumab and ipilimumab. New onset diabetes mellitus was diagnosed on the basis of elevated hemoglobin A1c, in the absence of prior personal or family history. Autoimmune or type 1 diabetes mellitus was ruled out with normal levels of anti-glutamic acid decarboxylase 65 (GAD65) antibody, zinc transporter 8 (ZnT8) antibody, and islet antigen-2 (IA-2) antibody.

**Conclusions:**

This case report highlights the importance of recognizing rare but serious adverse events related to immunotherapy and incorporation of appropriate tools for early identification and management in national cancer treatment guidelines.

## Background

Utilization of immunotherapy in the treatment of hematologic and oncologic disorders has grown exponentially over the last decade, with the number of diseases being treated continuing to grow. Given their wide use, many of the common adverse effects have already been recognized and incorporated into the adverse events management guidelines. However, there are some rare immune mediated effects that remain under-recognized and therefore pose a diagnostic challenge to clinicians. T cells, B cells and macrophages express programmed cell death-1 (PD-1) receptors, which negatively regulate immune responses by binding to their ligands programmed cell death ligands 1 and 2 (PD-L1 and PD-L2). Cancer cells evade the host immune system by expressing these ligands. Similarly cytotoxic T-lymphocyte-associated protein-4 (CTLA-4) is expressed by activated T cells, which acts as an immune checkpoint and downregulates immune responses against cancer cells. Immunotherapy, specifically, checkpoint inhibitor regimens such as nivolumab (anti-PD-1 monoclonal antibody) and ipilimumab (anti-CTLA-4 monoclonal antibody) target these receptors, thereby allowing the host immune system to mount a response against cancer cells.

Anti-PD-1 and anti-CTLA-4 agents have been linked to several autoimmune related side effects arising from T-cell activation. The incidence of autoimmune hypophysitis induced by anti-CTLA-4 monoclonal antibodies has varied from 0 to 17% of treated melanoma patients. [1] Nivolumab, an anti- PD-1 monoclonal antibody, is known to cause immune mediated side effects including pneumonitis, colitis, hepatitis, nephritis, and hypothyroidism. Apart from these, there also exists the likelihood of developing immune mediated new onset type 1 diabetes mellitus, which has been described in mice models as well as in humans. [2] This entity remains under recognized and currently is not part of the National Comprehensive Cancer Network’s (NCCN) and American Society of Clinical Oncology’s (ASCO) guidelines on management of immunotherapy related side effects. Only few cases of immunotherapy related fulminant diabetes mellitus have been reported so far. [3–5]

We present a case of a patient treated with combination checkpoint inhibitor therapy (ipilimumab and nivolumab) for metastatic melanoma, who presented with diabetic ketoacidosis (DKA) arising from new onset diabetes mellitus, initially thought to be autoimmune related. But, the autoimmune biomarkers returned negative pointing towards type 2 diabetes mellitus related DKA.

## Case presentation

A 42-year-old man with no other significant medical history was diagnosed with metastatic melanoma that was BRAF V617F mutated, with metastasis to liver, lung and adrenal glands. He had a past history of early stage melanoma that was initially diagnosed eight years ago, for which he underwent wide local excision with a negative sentinel lymph node biopsy. He did not receive any adjuvant chemo or immunotherapy. Subsequently, he started noticing multiple cutaneous lesions that were positive for melanoma, which led to a complete staging work up that revealed metastatic disease. Patient had an excellent performance status with no known history of endocrinopathies, including diabetes mellitus. He had normal fasting glucose levels, which was checked by his primary care physician. He was started on first line systemic immunotherapy with the combination of ipilimumab and nivolumab. He completed three out of the four planned cycles of combined regimen, that was administered at ipilimumab 3 mg/kg IV and nivolumab 1 mg/kg IV every three weeks, prior to emergency room presentation. Chief complaints at this presentation included intractable nausea, vomiting and diarrhea. He reported to having more than 8 loose bowel movements a day, some of which were associated with blood streaking. In the ER his serum glucose was elevated to 728 mg/dL (Table 1) and he was in DKA with significant anion gap metabolic acidosis, for which he was admitted to intensive care unit for further management. He was given intravenous insulin as bolus and started on insulin drip along with IV fluids as per DKA protocol. His blood glucose levels subsequently improved. Hemoglobin A1c (HbA1c) level from admission was 6.5%, indicating a rather new onset diabetes mellitus. Stool studies returned negative for infectious etiologies and he was started on anti-motility agents (Imodium and Lomotil), which failed to provide any relief from diarrhea. Computerized tomography of abdomen and pelvis showed pan-colitis and he was started on prednisone 1 mg/kg daily for presumed immune mediated colitis. Despite steroids he continued to have diarrhea, which were intermittently bloody. He was then started on octreotide (50 mcg subcutaneous injection TID, which was later increased to 100 mcg TID), with which his diarrhea was controlled. We had planned to start him on infliximab (a tumor necrosis factor inhibitor) if he failed the octreotide trial.

**Table 1:** Complete Metabolic Panel on presentation

Sodium	127 mmol/L
Potassium	5.7 mmol/L
Chloride	88 mmol/L
Bicarbonate	14 mmol/L
BUN	27 mg/dL
Creatinine	1.5 mg/dL
Glucose	728 mg/dL
Calcium	10.8 mg/dL
Total Bilirubin	0.5 mg/dL
Alkaline Phosphatase	128 U/L
AST	13 U/L
ALT	27 U/L
Total Protein	8.9 g/dL
Albumin	4.5 g/dL
Anion Gap	25

Subsequently, he was discharged home on an insulin regimen for presumed new onset insulin dependent diabetes mellitus (IDDM) and prednisone taper for colitis. Initially he had trouble controlling blood glucose levels while on prednisone taper. But, once he was off prednisone, his IDDM was better controlled. One month later into follow up repeat hemoglobin A1c was 7.9%, but his glucose levels were much better controlled. Since no testing for autoimmune diabetes was done during his initial presentation, anti-GAD65 antibody, ZnT8 antibody, and IA-2 antibody testing was done during his subsequent clinic follow up. Anti- GAD65 antibody was <5 IU/ml, ZnT8 antibody was <10 U/Ml, and IA-2 antibody was <0.8 U/ml, all being within normal limits. Though DKA is more common with autoimmune or type 1 diabetes mellitus, it can be seen with type 2 diabetes mellitus. Since, patient had no evidence of diabetes or pre-diabetes prior to immunotherapy, we think treatment with combined checkpoint blockade is what led to DKA.

## Discussion and conclusions

IDDM associated with immunotherapy has been reported with non-checkpoint inhibitor agents like high dose interleukin-2 (IL-2) and interferon-alpha (IFN-α). [6, 7] New onset IDDM associated with the use of newer immunotherapy agents such as nivolumab and pembrolizumab (checkpoint inhibitors) has been documented in case reports. [4] Ipilimumab was shown to be associated with the onset of other endocrinopathies like thyroiditis, hypophysitis and adrenal insufficiency, but not so much in terms of new onset diabetes mellitus, especially type 2. [1] In metastatic melanoma use of combined checkpoint inhibitor therapy (nivolumab in combination with ipilimumab) has been shown to increase overall survival. Though there is not much information available on incidence of IDDM with the use of CTLA-4 inhibitors in humans, mouse model studies have shed some light on the mechanisms involved with induction of insulitis and its progression to diabetes. [8, 9] Non-obese diabetic mouse model studies have suggested CD28/B7/CTLA-4 co-stimulatory pathway to be important for induction of insulitis, whereas PD-1/PD-L1 pathway is involved with regulation of both induction of insulitis and progression into autoimmune diabetes. This could have explained the physiologic basis for incidence of new onset of diabetes in our patient, a process that is regulated by auto reactive T-cells that target and destroy pancreatic beta cells. However, subsequent negative autoimmune work up indicates this being a non-autoimmune phenomenon. In our review of literature, we found some case reports that demonstrated incidence of DKA starting as early as 1 week and ranging up to 5 months after beginning treatment with nivolumab and pembrolizumab. [3, 4] Our index case also falls into this time period since the patient was diagnosed with DKA about 3 months after initiation of combination checkpoint inhibitor therapy, which led to our initial assumption that it was indeed IDDM. Unfortunately human leukocyte antigen (HLA) typing, anti-GAD65, IA-2 and ZnT8 antibody testing was not obtained as part of the initial workup, which are useful in the diagnosis of IDDM, nor did he have any prior testing done before initiation of immunotherapy. There was a recently published case report of autoimmune diabetes being diagnosed in the setting of nivolumab treatment for non-small cell lung cancer. However, in that case, testing was performed on pre-treatment frozen blood samples that showed elevated biomarkers despite of there being no other signs or symptoms of diabetes mellitus. [10] It was inferred that initiation of immunotherapy may have exacerbated underlying autoimmune diabetes, leading to DKA. Our patient had no prior diagnosis or testing performed for diabetes and his autoimmune biomarkers were within normal limits. However, at presentation with DKA his HbA1c was 6.5%. He had no prior abnormal fasting or postprandial glucose levels, nor did he have a baseline HbA1c for comparison. This patient was a fit, non-obese male with no other comorbidities or significant family history of diabetes mellitus. Prior to beginning his immunotherapy his thyroid-stimulating hormone, cortisol and adrenocorticotrophic hormone levels were all within normal limits.

Checkpoint inhibitor immunotherapy has proven its mettle in improving survival in various solid malignancies and continues to expand its territory to include hematologic malignancies. With the ever-increasing utilization of immunotherapies in the current era of cancer treatments, recognition of rare but serious adverse events like type 1 or 2 diabetes mellitus which can manifest as DKA, carries great importance. This requires a multidisciplinary approach starting with proper patient education prior to treatment initiation, close monitoring of appropriate biomarkers or tests with a low level of suspicion along with incorporation of appropriate tools for early diagnosis and proper management of these adverse events in the national cancer therapy guidelines.

## Abbreviations

anti-GAD65 antibody: Anti-glutamic acid decarboxylase 65 antibody
ASCO: American Society of Clinical Oncology
CD28: Cluster of differentiation 28
CTLA-4: Cytotoxic T-lymphocyte-associated protein-4
DKA: Diabetic ketoacidosis
IA-2 antibody: Islet antigen-2 antibody
IDDM: Insulin-dependent diabetes mellitus
NCCN: National Comprehensive Cancer Network
PD-1: Programmed cell death-1
PD-L1: Programmed cell death-ligand 1
PD-L2: Programmed cell death-ligand 2
TID: Three times a day
ZnT8 antibody: Zinc transporter 8 antibody
